# Comparative results of endoscopic and open methods of vein harvesting for coronary artery bypass grafting: a prospective randomized parallel-group trial

**DOI:** 10.1186/s13019-015-0353-3

**Published:** 2015-11-12

**Authors:** Alexander Chernyavskiy, Alexander Volkov, Oleg Lavrenyuk, Igor Terekhov, Yulia Kareva

**Affiliations:** 1 Novosibirsk Research Institute of Circulation Pathology, Rechkunovskaja str. 15, 630055 Novosibirsk, Russia; 2 Stroiteley str., 9, 46, Novosibirsk, Russia

**Keywords:** Endoscopic vein harvesting, Coronary artery bypass surgery

## Abstract

**Background:**

We compared wound complications between endoscopic and open great saphenous vein harvesting for coronary artery bypass surgery.

**Methods:**

A total of 228 consecutive patients were prospectively randomized into two groups: open vein harvesting (OVH), 115 patients; and endoscopic vein harvesting (EVH), 113 patients. Each group was assessed for post-operative wound complications, pain intensity, and neuropathy in the early post-surgical period. Lymphoscintigraphy of the lower limbs as well as morphological studies of vein walls using light and electron scanning microscopy were performed.

**Results:**

Vein harvesting time was shorter for EVH than OVH: 31.8 ± 6.2 min and 40.3 ± 15.8 min, respectively (*p* < 0.01). There were fewer complications after vein harvesting in the EVH group (11.5 %) than in the OVH group (44.4 %) (*р* = 0.001). Multivariate analysis showed that diabetes mellitus was the only risk factor for post-surgical complications after OVH (odds ratio = 3.95 %; 95 % confidence interval 1.03–8.6). Lymphoscintigraphic data in the EVH group did not demonstrate considerable disturbances in lymph drainage after surgery. In the OVH group, the accumulation of radiopharmaceutical drugs in the lymphatic nodes reduced two-fold (*р* ≤ 0.001). Histological evaluation of vein samples did not show considerable damage to the vein wall in either group.

**Conclusions:**

Using electron microscopy of vein fragments, this study demonstrated that EVH reduces wound complications and provides good-quality conduits.

## Background

The great saphenous vein (GSV) remains one of the most commonly used conduits due to its ease of harvesting, availability, and versatility [1]. Traditional harvesting of the GSV involves the open-vein technique, which requires an extended leg incision. This technique is associated with a significant morbidity rate, and wound complications occur in 2–24 % of cases [2, 3].

Minimally invasive techniques such as endoscopic vein harvesting (EVH) have therefore been developed to reduce post-coronary artery bypass grafting (CABG) leg wound complications. Currently, EVH is the method of choice in many centers as it allows lower post-surgical complication rates compared to the open method [2, 4, 5]. Although long-term graft patency following EVH has been questioned [6], cohort studies have reported that the technique is safe [7] and effective.

The possibility of using lymphoscintigraphy to evaluate the lymphatic system of the lower limb after vein harvesting for coronary artery bypass surgery has been reported previously [8]. Nevertheless, the state of the lymphatic system after vein harvesting is still poorly studied.

Currently, there is no consensus regarding the integrity and quality of the conduit following vein harvesting, which can have various impacts on the vein wall [9, 10]. With this in mind, we studied the initial state of the venous conduit, peri-operative vein damage, and post-operative wound complications using two methods of GSV harvesting.

## Methods

The present study was designed as a prospective, parallel-group trial to assess wound complications in two groups of patients. The study was approved by the local ethics committee, and conducted from 2010 to 2012, in compliance with the approved protocol and in accordance with standard operating procedures. Informed consent was obtained from all patients in accordance with our institutional research ethics review board guidelines. The study included 228 patients diagnosed with ischemic heart disease who underwent coronary artery bypass surgery from 2010 to 2012 at the Novosibirsk Research Institute of Circulation Pathology. The primary study end point was to identify differences in the clinical and functional condition of the lower limbs after the two methods of vein harvesting. The sample size required to address the primary end point was calculated on the basis of differences in lower limb wound complications in previous studies involving patients treated with EVH and OVH [11]. Assuming a 15 % improvement with the EVH method and applying the variance seen in our patients, we anticipated a sample size of 113 per group to demonstrate a significant difference (*p* < 0.05) at a power of 90 % and with a 5 % dropout rate.

Randomization to either the open vein harvesting (OVH) group (*n* = 115) or the EVH group (*n* = 113) was performing in blocks of 10 with an allocation ratio of 1:1 using sequentially numbered, opaque, sealed envelopes. The designated study coordinator, who was not involving in filed procedures, was responsible for the preparation of the randomization list.

Inclusion criteria: subjects with multivascular lesions of the coronary artery who were suitable for coronary artery bypass surgery.

Exclusion criteria: patients requiring urgent coronary artery bypass surgery with unstable hemodynamics; previous coronary artery bypass surgery; chronic venous insufficiency С4–С6 according to the СЕAP classification; and previous limb surgery.

All veins were harvested by experienced surgeons with previous experience of more than 100 procedures using both OVH and EVH.

Wound assessment was completed daily by a specialist wound nurse and a research team for the first 7 days after discharge.

### Open vein harvesting

OVH was performed using a continuous incision under direct visualization. The GSV was identified two-fingers proximal to the medial malleolus according to standard practice. The vein was harvested using Metzenbaum scissors, and a continuous incision was made along the route of the vein. Care was taken not to traumatize the nerve, vein, or its branches. Vein branches were ligated with titanium clips. The wound was closed in layers with continuous 2–0 Polysorb sutures and 3–0 skin sutures. Immediately after vein harvesting, the lower leg was tightly bandaged, and elastic stockings were used in all patients after the operation.

### Endoscopic vein harvesting

EVH was performed through minimal incisions using a Vasoview 6 system with CO_2_ insufflation into the closed cavity.

The vein was identified on the medial tibial border through a 3-cm incision just below the knee as per standard practice. It is significantly easier to harvest vein conduits using EVH from the thigh due to the size and positioning of the endoscopic equipment and hence the ease of access to the vein. The incision site was sealed using a balloon port to create a tunnel inside the leg. CO_2_ insufflation was then commenced at 12 mmHg of pressure with a 3 L/min flow rate. A dissection tip cannula was introduced inside the tunnel to isolate the vein and its branches. A second unit with cautery was inserted via the port to cut and seal the tributary branches. A 1-cm skin incision was made near the groin to ligate the distal end of the GSV and remove the vein, which was checked for leakage. The branches were tied with titanium clips, and necessary repairs were performed using 7–0 Prolene sutures. The wound was closed with 3–0 skin sutures. Immediately after vein harvesting, the lower leg was tightly bandaged, and elastic stockings were used in all patients after the operation.

After vein harvesting, a standard CABG procedure (cardiopulmonary bypass and cardioplegia) was performed.

The analysis of intraoperative data included: vein harvesting time point (total vein harvesting time - total time spent harvesting the vein, preparation of the vein for bypass surgery, and suturing wound time; vein harvesting time - only the time spent harvesting the vein, without preparation time and time spent suturing the wound; vein preparation time - the time spent on clipping the inflows to the veins and the closure of defect), wound closure time, harvested conduit length, and post-operational incision length on the lower limb. Assessment of the clinical state of the limb in the early post-surgical period included the following factors: wound healing and the frequency and intensity of post-surgical complications. In order to identify post-operational edema of the lower limb, the circumference at three levels before surgery and on day 7 following surgery was measured. Post-operative lymphedema was defined as an apparent increase in limb volume compared to the pre-operative status accompanied by induration of the surrounding tissues. Lymphorrhea was defined as the leakage of lymph from the wound after surgery. Pain intensity in the lower limb was established on the basis of a digital rating scale from 0 to 10 (0 = no pain to 10 = unbearable pain).

Evaluation of the initial and post-operative state of the lymphatic drainage in the lower limb was performed on the basis of radionuclide lymphoscintigraphy in 41 patients (OVH = 18 [43.9 %] patients, EVH = 23 [56.1 %] patients).

Lymphoscintigraphy was performed on the day before surgery and on day 7 after surgery. Drug evacuation from the inguinal lymphatic nodes was assessed 1 h after the patient began walking. In order to evaluate the resulting images, a scintigram point system was developed depending on the degree of disturbances from Grade 0 (no disturbances) to Grade 3 (expressed disturbances).

Morphostructural evaluation of the wall state of the GSV was performed in 161 patients (OVH = 83 [51.6 %], EVH = 78 [48.4 %]) using light and scanning electron microscopy. The GSV samples were used as materials for histopathological studies. Vein fragments with dimensions of 5–7 mm were immobilized in 10 % formalin solution. Five μm thick sections were stained using hematoxylin and eosin and van Gieson’s stain with additional staining of elastic fibers by Weigert’s resorcin-fuchsin. The endothelial lining was studied by scanning electron microscopy. The uniformity of the endothelial layer, deformation and desquamation of endothelium and polymorphism, and platelet reactivity were assessed.

### Statistical analysis

Statistical analysis was performed with Statistica 6.0 software (Statsoft, USA). Results were shown as mean values (± standard deviation) for quantitative values or as values and percentages for qualitative values. In order to detect dependence between the studied parameters, we performed the Spearman ratio of rank correlation as well as the odds ratio (OR) and absolute risk calculation. In order to calculate odds ratios, the 95 % confidence interval (CI) was calculated. *p*-values < 0.05 were considered statistically significant.

## Results

The groups did not differ with respect to most of the studied pre-operative characteristics except for atherosclerosis of the lower extremities, which was observed more frequently in the EVH group (20 patients [17.7 %]) than in the OVH group (8,7 %), *p* = 0.008 (Table 1).

**Table 1:** Comparative subject characterization

Parameters	OVH (*n* = 115)	EVH (*n* = 113)	*р*
Male, n(%)	95(82.6 %)	93(82.3 %)	0.169
Age, years	60 ± 8.1	61 ± 7.4	0.951
CCS-class	2.8 ± 0.68	2.9 ± 0.84	0.49
NYHA-class	2.8 ± 0.7	2.8 ± 0.83	0.83
Weight, kg	85.7 ± 14.1	82.9 ± 15.2	0.107
Height, cm	169.6 ± 7.8	168.1 ± 8.2	0.130
Body weight index, kg/m^2^	29.70 ± 3.88	29.06 ± 4.81	0.181
Obesity^a^[n (%)]	14 (12.2)	14 (12.4)	0.450
Diabetes mellitus [n (%)]	20 (17.4)	21 (18.6	0.323
Lower limb atherosclerosis [n (%)]	10 (8.7)	20 (17.7)	0.008
Arterial hypertension [n (%)]	109 (94.8)	95 (84.1)	0.075
LV EF (%)	55 ± 13.66	55.6 ± 13.67	0.82
EuroSCORE	5.5 ± 0.7	5.6 ± 2.2	0.65

*CCS* Canadian Cardiovascular Society grading of angina pectoris, *LVEF* left ventricular ejection fraction, *NYHA* New York Heart Association;

Note – ^a^by BWI – for males >32, for females >34

No cases of conversion to OVH were reported during the study. The average number of bypassed coronary arteries in the OVH group was 2.7 ± 0.6, whereas in the EVH group it was 2.8 ± 0.6 (*p* = 0.117). The intra-operative data of patients from both groups are shown in Table 2. As can be seen from the table, almost all of the studied parameters were different between the two groups.

**Table 2:** Intraoperative results of great saphenous vein harvesting

Parameters	OVH (*n* = 115)	EVH (*n* = 113)	*р*
	М ± SD	М ± SD	
Total vein harvesting time, min	40.3 ± 15.8	31.8 ± 6.2	0.01
Vein harvesting time, min	18.3 ± 7.6	24.7 ± 5.5	0.03
Post-surgical wound closure time, min	21.1 ± 8.5	3.5 ± 0.7	0.002
Time of vein preparation, min	0.9 ± 1.5	3.7 ± 1.3	0.03
Harvested venous conduit length, cm	31.6 ± 9.4	37.3 ± 4.2	0.04
Skin incision length, cm	35.4 ± 10.2	6.3 ± 0.9	0.001

Assessment of post-surgical complication frequencies revealed that the total number of patients with post-operative complications was 44.4 % in the OVH and 11.5 % in the EVH group (*p* < 0.01) (Table 3). Post- operative complications were more frequent in the OVH group, especially lymphorrhea (*р* = 0.033), lymphedema (*р* = 0.001), post-surgical diastasis (*р* = 0.046), paresthesia (*р* = 0.003), and inflammatory changes (*р* = 0.011).

**Table 3:** Postoperative complications

Parameters	OVH (*n* = 115)	EVH (*n* = 113)	*р*
Hematoma	2 (1.7 %)	4 (3.5 %)	0.397
Lymphorrhea	7 (6.1 %)	1 (0.9 %)	0.033
Postoperative lymphedema	36 (31.3 %)	4 (3.5 %)	0.001
Skin diastasis	4 (3.5 %)	0 (0 %)	0.046
Post-surgical wound infection	1 (0.9 %)	0 (0 %)	0.322
Paresthesia	17 (14.8 %)	4 (3.5 %)	0.003
Soft tissue inflammation	15 (13.0 %)	5 (4.4 %)	0.011
Number of subjects with complications	51 (44.4 %)	13 (11.5 %)	0.001

Correlation analysis in the OVH group detected statistically significant relationships between complications and diabetes mellitus (*p* = 0.04). Regression analysis of post-surgical complications in the OVH group identified diabetes mellitus as the only risk factor. Diabetes mellitus increased the probability of complications by three-fold (OR = 3, 95 % CI 1.03–8.6).

There were statistically significant differences in the levels of pain experienced between patients in the two groups in the post-operative period. In the EVH group, 47.8 % of subjects experienced no pain (0 points), compared to only 28.7 % of patients in the OVH group (*p* < 0.003). The probability of experiencing pain in the OVH group during the post-operative period was 2.3 times higher than in the EVH group (OR = 2.3, 95 % CI 1.3–3.9, *p* < 0.05).

Radionuclide lymphoscintigraphy showed that in the EVH group, the accumulation of radioactive drugs in the inguinal lymphatic nodes of the limb reached 17.87 ± 11.95, and 16.22 ± 9.75 after surgery (*p* = 0.34). In the OVH group, the accumulation of radioactive drugs was somewhat higher, reaching 20.89 ± 11.53. The lymphoscintigraphy findings on day 7 after surgery showed a reduction in radioactive drug accumulation (9.78 ± 7.02, *p* ≤ 0.001).

**Fig. 1:**
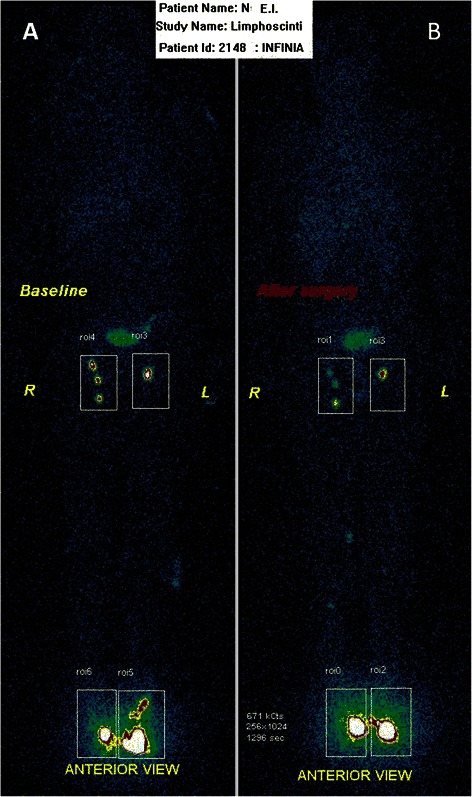
Lymphoscintigrams before the surgery (**а**) and on Day 7 after the endoscopic vein harvesting on the right lower limb (**b**)

According to the pre-surgical lymphoscintigraphy data, there were two patients in the EVH group with an insignificant degree of lymphatic drainage failure, while two patients in the OVH group had similar changes. In the post-operative period in the EVH group (*n* = 23), there were eight (34.8 %) patients with lymphatic drainage dysfunction (seven [30.4 %] insignificant, one [4.3 %] moderate), while in the OVH group there were 17 (94.4 %) patients with similar issues (four [22.2 %] insignificant, five [27.7 %] moderate, eight [44.4 %] severe) (Fig. 1).

**Fig. 2:**
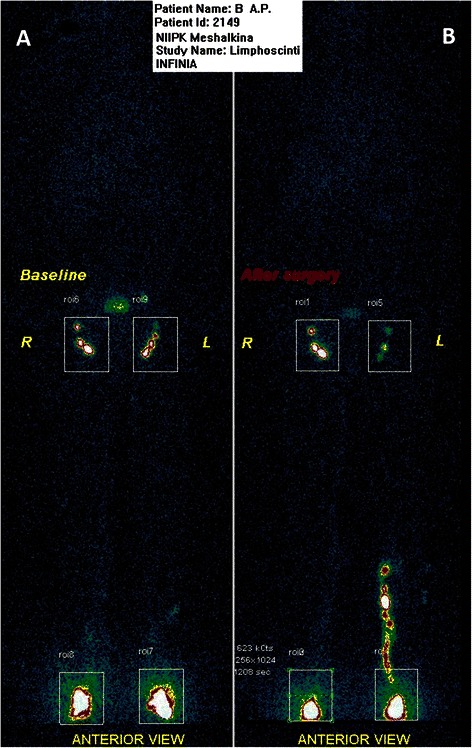
Lymphoscintigrams: before the surgery (**а**) and on Day 7 after the open vein harvesting on the left lower limb (**b**)

In the OVH group (*n* = 18) after surgery, an insignificant degree of change was observed in four (22.2 %) patients, moderate change in five (27.8 %), and severe change in eight (44.4 %) (Fig. 2).

Thus, the total numbers of patients with lymphatic drainage disturbances in the OVH and EVH groups were 17 (94.4 %) and eight (34.8 %), respectively (*р* = 0.001).

When comparing lymphoscintigraphy data and clinical manifestations of lymphatic drainage disturbances after surgery (early lymphatic drainage and lymphorrhea), deviations were detected in nine patients (50 %) in the OVH group (*n* = 18) (seven cases of lymphatic drainage and two of lymphorrhea). In the EVH group (*n* = 23), these complications were not detected in any patients.

The data on structural damage of the vein wall are presented in Table 4. Focal endothelial layer desquamation predominantly along the length from 20 to 250 μm was detected in 30 cases (36.1 %) in the OVH group and in 23 cases (29.4 %) in the EVH group (*p* = 0.37). Intimal ruptures occurred in the fibrous-thickened internal later in the perpendicular-longitudinal and circular-longitudinal directions relative to the vein lumen axis (23 patients [27.7 %] in the OVH group compared to 19 [24.4 %] in the EVH group, *р* = 0.63).

**Table 4:** Types of structural vein wall damages

Types of GSV damages	OVH (*n* = 83)	EVH (*n* = 78)	*р*
Focal endothelial layer desquamation	30 (36.2 %)	23 (29.5 %)	0.37
Vein wall dissection (without superficial damage)	0 (0 %)	5 (6.4 %)	0.02
Vertical and horizontal ruptures of the internal layer	23 (27.7 %)	19 (24.4 %)	0.63
Focal and diffuse edema of the vein wall	37 (44.6 %)	32 (41 %)	0.65
Adhesion of corpuscles to the endothelial surface	10 (12.1 %)	12 (15.4 %)	0.54
Parietal thrombi development	0 (0 %)	1 (1.3 %)	0.30
Paravascular soft tissue coagulation	0 (0 %)	7 (8.9 %)	0.004
Total GSV samples with identified damages	45 (54.2 %)	47 (60.3 %)	0.45

Vein dissection was isolated and predominantly related to EVH cases (5 [6.4 %], *р* = 0.02).

Vein vascular wall changes were rarely isolated, and more often were accompanied by other damage such as rupture or dissection (37 patients [44.6 %] in the OVH group compared with 32 [41 %] in the EVH group, *р* = 0.65). The EVH method included coagulation of the vein branches; therefore, specific changes in the vein wall, such as perivascular soft tissue coagulation, were detected by histopathological analysis. Coagulation of paravascular tissue was observed in just seven (8.9 %) patients in the EVH group (*р* = 0.004).

Correlation analysis showed direct dependence between initial changes of the GSV and the extent of damage during OVH (*p* = 0.03) and EVH (*p* = 0.02), which allowed us to determine the risk of damage in the event of mild initial changes (AR = 32 %, 95 % CI 20–44), moderate changes (АR = 75 %, 95 % CI 63–89), and severe changes (АR = 87 %, 95 % CI 77–97).

## Discussion

CO_2_ insufflation is used during EVH to create a closed working tunnel for vein preparation and harvesting. The recommended CO_2_ pressure is between 10 and 15 mmHg. Complications caused by CO_2_ insufflation are rare. Chiu et al. showed that significantly more CO_2_ can be detected by transesophageal echo in the inferior cava during EVH with a working pressure of 15 mmHg [12–15]. Knowing this, we did not exceed a pressure of 10–12 mmHg. Therefore, specific complications associated with CO_2_ insufflation were not observed in this study, although we did not carry out specific examinations to identify them (e.g. transesophageal echocardiography).

The obtained data demonstrated that the method of vein harvesting used has a significant impact on the post-surgical wound complications experienced by patients, which is in accordance with the results of other studies [4, 16, 17]. Fewer wound complications were observed in the EVH group, with an absence of significant differences between the two groups according to the frequency of obesity and diabetes mellitus. At the same time, diabetes mellitus was an independent risk factor of post-surgical complications in the OVH group.

According to the results of the present study, the risk of paresthesia in the lower limb increased by 4.7 times in the OVH group. The higher percentage of complications in the OVH than the EVH group was accounted for by more frequent early lymphatic drainage. Thus, in the EVH group, according to the findings of previous studies, lymphatic drainage was observed in approximately 10–12 % of patients [18]. However, in the present study, it was only observed in 4.5 % of patients. The number of cases of lymphorrhea was higher (6.1 %) in the OVH group.

Another important aspect of surgical therapy is the degree of pain experienced by the patient after surgery. For patients, the reduction of post-surgical pain is the best predictor of satisfaction with surgery. Our study showed significantly lower intensities of post-surgical pain in the EVH group than in the OVH group, which is in accordance with the results of other studies.

Immediate patient satisfaction is important when assessing vein harvesting techniques. However, conduit quality and prognostic implications must be the primary outcomes. EVH required more frequent vein repairs than OVH. Minimally invasive vein harvesting techniques reduce visualization of the graft, and are more technically complex. This can potentially lead to reduced vessel integrity. Given the recent finding by Lopes et al. [6] that EVH may be associated with reduced graft patency, this represents a potentially important finding. However, another large cohort study reported no mid-term effect on mortality associated with EVH [7]. Although bridging technique was associated with more minor repairs than OVH, it requires thorough long-term follow-up and provides equivalent long-term outcomes [19]. This indicates that reduced vessel integrity and subsequent vein repair do not affect clinical outcomes, although a formal histological assessment of this hypothesis is required.

In our study, we performed histological examination of vein fragments after both techniques. Morphological studies revealed general wall damage types during venous autogenous graft collection as well as specific issues associated with EVH. The following factors were identified: coagulation of paravascular soft tissues associated with the EVH method, and wall dissection accompanied by layer displacement along the longitudinal axis of the vessel.

In addition to endothelial layer damage, light microscopy revealed a number of other structural manifestations. The most frequent of these was corpuscle adhesion to the de-endothelialized surface. This effect might have been the result of damage to the endothelial layer with the development of high thrombogenicity of the subendothelial tissue to circulating platelets. However, taking into account that the anti-thrombotic properties of the vein are comparatively low, there is a high probability of clotting that needs to be prevented by the introduction of heparin during the vein harvesting stage [20].

We evaluated lymphatic drainage in the lower limbs by radionuclide lymphoscintigraphy. We demonstrated the effect of incision length during OVH on damage to the lymphatic system of the lower limb. Clinical manifestations of lymphatic drainage in the early post-surgical period depend directly on damage to lymphatic drainage function. The aggravation of these clinical manifestations might be accounted for pre-clinical lymphatic drainage function. Thus, lower limb lymphoscintigraphy in the early post-surgical period allows the detection of patients at risk for lymphedema during vein harvesting. A previous study [9] showed that by the end of a 5-year observation period following GSV harvesting, lower limb drainage manifestations increased by more than two-fold (reaching 46.1 %) compared to those observed at the 2-year observation period. The present study demonstrated that GSV harvesting for CABG causes various degrees of disturbances to the functional state of the lower limb, and that the intensity of the impact on lymphatic drainage function directly depends on the harvesting method used.

The limitations of this study are the single-center design, the short duration of follow up, and the small number cases in which lymphatic drainage in the lower limb was evaluated. Only 41 patients were assessed (OVH = 18, EVH = 23 patients). In our study, vein harvesting was carried out at different sites of the lower limb in each group (from the leg in the OVH group and the hip in the EVH group). This is another limitation, but we do not consider it to be serious because tissue damage in the thigh caused by the open method is obviously more traumatic than damage caused by vein harvesting in the leg. Therefore, in our opinion, it is unethical to subject patients to what is known to be a traumatic procedure if it can be avoided. Despite the different techniques of vein harvesting used in both groups, wound closure was performed using cosmetic skin suturing. In our study, we evaluated the early results of operations using the two methods of vein harvesting, such as wound complications, lymphatic drainage of the lower extremities, and histological status of the veins. We did not evaluate the patency of grafts in the long term despite the fact that this is a very important end point of the procedure. It is a limitation of our study and requires further evaluation in the future.

## Conclusion

The data obtained in this study confirm the effectiveness of EVH. GSVs harvested using the endoscopic technique are of comparable quality to those obtained using the open technique. These results were confirmed by histological studies. We demonstrated that EVH reduces the frequency of post-surgical complications and pain in lower limb wounds. At the same time, the impact on the lymphatic system of the lower limbs is significantly lower if the vein is harvested using endoscopic rather than open technique.

## Abbreviations

AR: Absolute risk
CEAP: clinical classification of chronic venous disorders
CI: Confidence Interval
EVH: Endoscopic vein harvest method
GSV: Great saphenous vein
OR: Odds ratio
OVH: Open vein harvest method
RPD: Radio pharmaceutical drug
Rs: Spearman ratio of rank correlation
